# MiR-21 is required for efficient kidney regeneration in fish

**DOI:** 10.1186/s12861-015-0089-2

**Published:** 2015-11-17

**Authors:** Beate Hoppe, Stefan Pietsch, Martin Franke, Sven Engel, Marco Groth, Matthias Platzer, Christoph Englert

**Affiliations:** Molecular Genetics Laboratory, Leibniz Institute on Aging - Fritz Lipmann Institute (FLI), Beutenbergstrasse 11, 07745 Jena, Germany; Genome Analysis, Leibniz Institute on Aging - Fritz Lipmann Institute (FLI), Beutenbergstrasse 11, 07745 Jena, Germany; Faculty of Biology and Pharmacy, Friedrich Schiller University of Jena, Fürstengraben 1, 07743 Jena, Germany; Present address: Research Group of Development & Disease, Max Planck Institute for Molecular Genetics, 14195 Berlin, Germany; Institute for Medical and Human Genetics, Charité Universitätsmedizin Berlin, 13353 Berlin, Germany

**Keywords:** Kidney regeneration, LNA, miRNA, Teleost killifish, igfbp3, fosl1

## Abstract

**Background:**

Acute kidney injury in mammals, which is caused by cardiovascular diseases or the administration of antibiotics with nephrotoxic side-effects is a life-threatening disease, since loss of nephrons is irreversible in mammals. In contrast, fish are able to generate new nephrons even in adulthood and thus provide a good model to study renal tubular regeneration.

**Results:**

Here, we investigated the early response after gentamicin-induced renal injury, using the short-lived killifish *Nothobranchius furzeri*. A set of microRNAs was differentially expressed after renal damage, among them *miR-21*, which was up-regulated. A locked nucleic acid-modified antimiR-21 efficiently knocked down miR-21 activity and caused a lag in the proliferative response, enhanced apoptosis and an overall delay in regeneration. Transcriptome profiling identified apoptosis as a process that was significantly affected upon antimiR-21 administration. Together with functional data this suggests that miR-21 acts as a pro-proliferative and anti-apoptotic factor in the context of kidney regeneration in fish. Possible downstream candidate genes that mediate its effect on proliferation and apoptosis include *igfbp3* and *fosl1,* among other genes.

**Conclusion:**

In summary, our findings extend the role of miR-21 in the kidney. For the first time we show its functional involvement in regeneration indicating that fast proliferation and reduced apoptosis are important for efficient renal tubular regeneration.

**Electronic supplementary material:**

The online version of this article (doi:10.1186/s12861-015-0089-2) contains supplementary material, which is available to authorized users.

## Background

Due to demographic change, kidney diseases have become a major health problem and the number of people suffering from acute or chronic kidney disease is increasing. Renal failure leads to reduction in the glomerular filtration rate and the loss of nephrons, the functional units of the kidney. Nephron structure is conserved among vertebrates and shows three main segments: glomerulus, proximal tubule and distal tubule. After renal injury mammals can regenerate segments such as proximal tubules and glomeruli partially, but are unable to form new nephrons and replace lost ones [1]. In contrast, in fish nephrogenesis and renal regeneration persists throughout life [2–4]. This is facilitated by a pool of progenitor cells, which are activated after induction of kidney injury, giving rise to new nephrons being visible as basophilic clusters of cells [3]. MicroRNAs are 20–22 nucleotides long non-coding RNAs and are known to play a role in several processes by regulating post-transcriptional steps of gene expression. While a role for miRNAs in regeneration of fish kidneys has not been demonstrated yet, a recent report shows a role for miR-34 in kidney morphogenesis [5]. In mammals a set of miRNAs was shown to be changed in its expression after kidney damage [6]. One of these miRNAs, *miR-21* is up-regulated after kidney injury [6, 7] and is involved in development of fibrosis [8]. In contrast to mammals, however, in fish only little or no scar formation accompanies the regenerative process as has recently been shown for the heart [9]. At the present time, the role of miRNAs in the regeneration of fish kidney has not been studied. Here, we have used the African killifish *Nothobranchius furzeri,* which has recently been established as a new model in aging research [10–12]. We have addressed the role of *miR-21* in the process of renal tubular regeneration and have found that it plays a critical role in kidney regeneration of *N. furzeri*.

## Results and discussion

### Neonephrogenesis and tubular regeneration in N. furzeri after renal injury

**Fig. 1:**
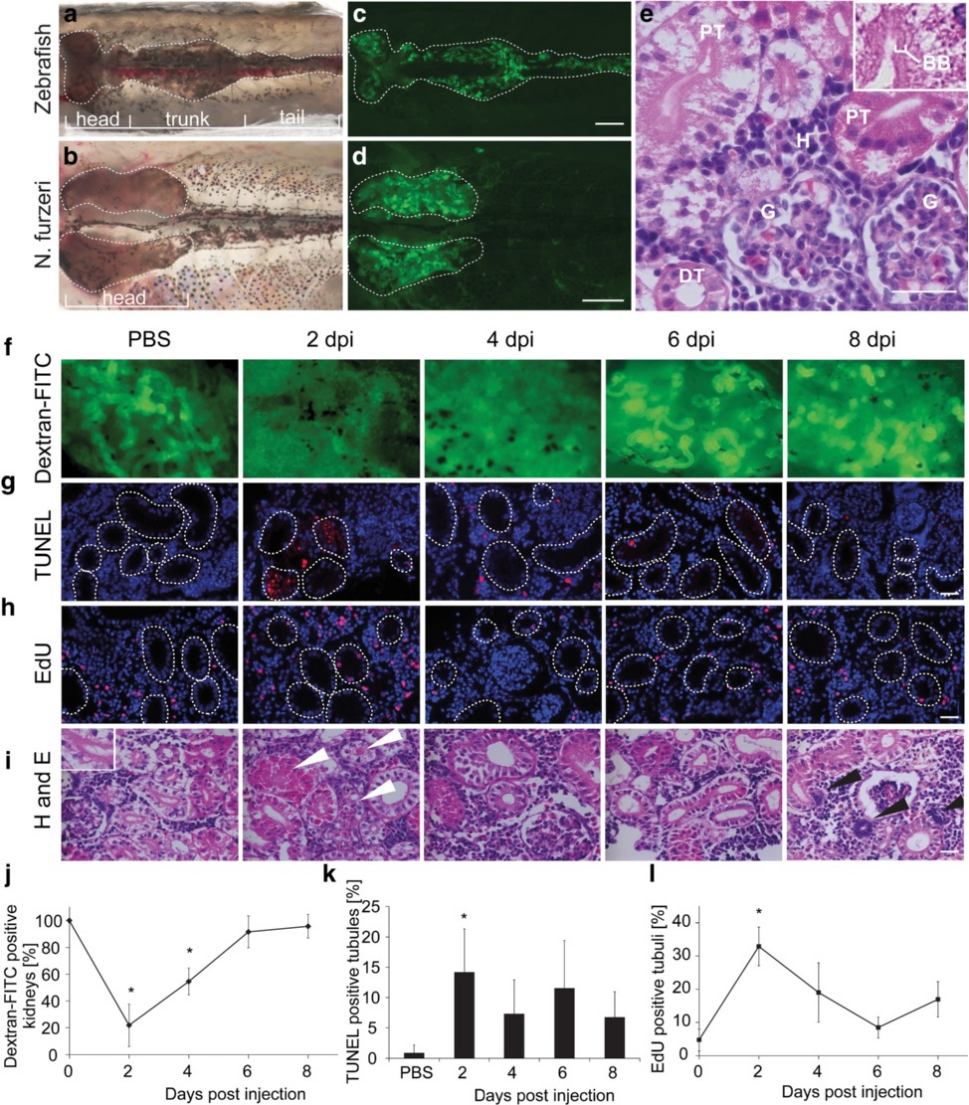
Damage of kidney by nephrotoxic gentamicin induces tubular regeneration and neonephrogenesis in *N. furzeri*. **a** Comparison of kidney anatomy of zebrafish and **b** *N. furzeri* after preparation and **c**, **d** after injection of fluorescent dextran-FITC, which is reabsorbed in the proximal parts of the tubule. Scale bar: 1 mm **e** Transverse section of *N. furzeri* kidney, stained with **h** and **e**. Different compartments of the nephron are indicated. Brush border, BB, of proximal tubule is shown in higher magnification. Abbreviations: G, glomerulus; PT, proximal tubule; DT, distal tubule; H, hematopoietic tissue. Scale bar: 50 μm. **f** After gentamicin application fish were injected with dextran-FITC 24 h prior to preparation every second day, to obtain information about recovery of kidney functionality after damage. **g** TUNEL-assay was performed to study apoptotic processes in kidneys after damage. Red color labels apoptotic cells, nuclei are labeled with DAPI in blue. **h** Fish were injected with EdU 2 h prior to being sacrificed. Encircled areas mark tubules in kidney of fish. Red staining shows incorporation of EdU into DNA, DAPI counterstaining is seen in blue. **i** H and E staining of kidneys after injection of PBS or gentamicin, *white arrows* label damaged tubules, *black arrows* indicate newly developing nephrons. Inset shows a tubule with an intact brush border. Scale bar **g**, **h**, **i**: 20 μm. Kidney function **j**, apoptotic cells **k** and proliferation **l** was quantified. To assess kidney function, all kidneys being positive for dextran-FITC were counted and related to all kidneys, *n* = 15 fish/time point. For quantification of apoptosis and proliferation, red labeled cells in tubules were counted and related to total number of tubules, *n* = 3–4 fish/time point

In fish two different kidneys can be found. While embryos and larvae possess a simple pronephros, consisting of one or two glomeruli, adult fish harbor a mesonephros. The latter can have different shapes, varying in respect to the size of the cranial and caudal portion [13–15]. This led us to investigate the kidney structure of *N. furzeri* and compare it to that of zebrafish (Fig. 1a and b). In zebrafish, the kidney is located at the dorsal side of the body and shows three different parts from anterior to posterior: head, trunk and tail kidney (Fig. 1a). While in *N. furzeri* the kidney is located at the dorsal side as well, it only shows an elongated head structure, thus resembling a head kidney only (Fig. 1b). This is comparable to the kidney of medaka [14]. Injection of 40 kDa dextran-FITC, a fluorescent sugar, being selectively reabsorbed in the proximal parts of the tubules confirmed the observations from bright field microscopy (Fig. 1c and d). Histological analysis of the *N. furzeri* kidney demonstrated the presence of glomeruli, proximal tubules (identified by brush border) and distal tubules as well as hematopoietic tissue (Fig. 1e). The presence of the latter is known form other fish species as well [16]. Having characterized the structure of the *N. furzeri* kidney, we next wanted to investigate renal tubular regeneration. After induction of kidney damage by intraperitoneal injection with the nephrotoxic drug gentamicin, which specifically damages the proximal parts of the tubules [17], the regeneration process was analyzed. Dextran-FITC was used to indicate kidney functionality, since it is no longer reabsorbed upon tubular damage [18] (Fig. 1f and j). Two days after administration of gentamicin, in 25 out of 32 fish (78 %) no dextran-FITC signal was seen, suggesting severe tubular damage. After 6 days, kidney function recovered and exhibited reabsorption of dextran-FITC. At 8 days post injection (dpi), a normal dextran-FITC signal was observed in 23 out of 24 fish (96 %). Compared to zebrafish, where tubular structure appears intact after 2 weeks post injury and functionality is restored after 3 weeks [19] recovery in *N. furzeri* is thus quite fast. We next wanted to examine the underlying cellular and molecular processes and first examined apoptosis and proliferation. Two days after damage induction, levels of apoptotic cells in the tubules increased to 14.2 % from less than 1 % prior injury (Fig. 1g and k). At 8 dpi levels of apoptotic cells in tubules dropped to 6.8 % indicating that the recovery process is not yet completely finished. Cell proliferation in the tubules was measured using an EdU-assay. At 2 dpi proliferating cells could be detected in 33 % of the tubules and decreased to basal level at 6 dpi (Fig. 1h and l). At 8 dpi proliferation again increased, however, the effect was not significant. To assess kidney damage and regeneration histologically, H&E staining was performed (Fig. 1i). In control kidneys, the brush border was found to be intact in proximal tubules. After gentamicin injection, cell aggregates were found in the lumen of proximal and distal tubules and the brush border was disrupted (Fig. 1i, white arrows). An enlargement of the lumen of tubules was observed 4 days post injection. Eight days post injection, specific basophilic structures were observed, indicating newly developing nephrons [20]. Combining functional and immunohistochemical data, we conclude that in *N. furzeri* initial and prompt repair processes take place in the tubules to allow fast functional recovery after kidney damage. Enhanced proliferation rates in the tubules at 2 and 4 dpi point towards regenerative processes in the tubules. Similar effects have been reported after renal injury in mammals [1], suggesting a similar response of tubular regeneration. In contrast to mammals, however, this process is followed by neonephrogenesis in fish.

### Differential expression of microRNAs after induction of kidney damage in N. furzeri

**Fig. 2:**
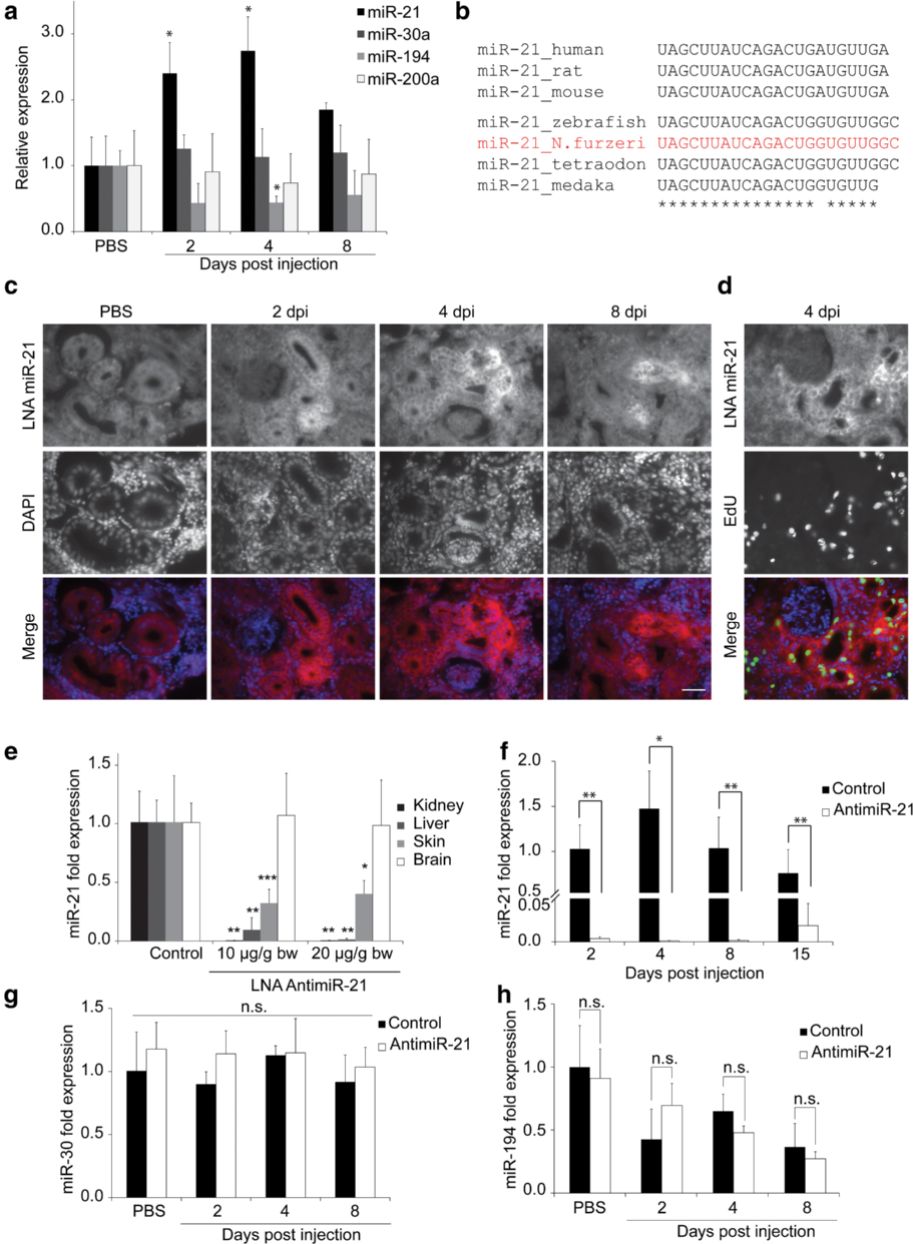
MiR-21 is up-regulated after renal injury and can be blocked specifically using LNA-antimiR-21. **a** Expression of miR-21, miR-30a, miR-194, miR-200a after induction of renal injury. MiRNA expression was measured using qRT-PCR. *n* = 4 fish/time point **b** Sequence analysis of miR-21 in different species. **c** In situ hybridization to show localization of miR-21 in fish kidneys at different time points after renal injury. For miR-21 detection an LNA-probe was used on kidney cryosections. Scale bar: 50 μm **d** EdU incorporation assay in combination with *in situ* hybridization to label proliferating cells and *miR-21* expression. **e** Analysis of *miR-21* expression after injection of antimiR-21 in two different concentrations and in four different tissues. *n* = 4 fish/time point **f** Time course of antimiR-21 stability in the kidney. **g** and **h** MiR-30 and miR-194 expression was measured by qRT-PCR in control and antimiR-21 treated kidneys. *n* = 4 fish/time point, one-way ANOVA or student’s *t*-test was used: *p < 0.05, **p < 0.01, ***p < 0.001

Previous reports have shown that a number of miRNAs are up- and down regulated after kidney damage in mammals [6, 21]. Whether these miRNAs also have an influence on kidney regeneration in fish has not been investigated yet. We selected a set of miRNAs with conserved expression in the kidney among vertebrates: miR-21, miR-30a, miR-194 and miR-200a [22]. Quantitative PCR (qPCR) for these miRNAs was performed using RNA from *N. furzeri* kidney tissues at 2, 4 and 8 dpi (Fig. 2a). After damage induction, *miR-21* was significantly up-regulated when compared to undamaged kidneys. *MiR-194* was down-regulated after induction of kidney damage, a result also seen in mammals [6]. *MiR-30a* and *miR-200a* expression levels were unchanged after renal injury. Previous studies have investigated the role of miR-21 in fibrosis following kidney damage in mammals; however, we did not observe fibrosis in fish after renal injury (Additional file 1: Figure S1). We therefore decided to study the influence of miR-21 on kidney regeneration in *N. furzeri*. Upon sequence comparison we found that miR-21 is highly conserved among mammals and fish (Fig. 2b). Especially the seed region is identical. In a next step, we investigated the localization of miR-21 in the kidney tissue. An *in situ* hybridization was performed with kidney cryosections of different time points after induction of kidney damage, using a locked nucleic acid (LNA) *in situ* probe (Fig. 2c). In undamaged control kidneys, *miR-21* was ubiquitously expressed in low amounts, in the hematopoietic tissue as well as in the kidney tubules. After damage induction, a significant and persistent increase of *miR-21* expression was found, especially in the tubules of the kidney. Interestingly, there was a significant overlap between *miR-21* positive and EdU positive cells suggesting a connection between *miR-21* expression and proliferation (Fig. 2d). These data are consistent with the qPCR results and show that miR-21 is specifically up-regulated in the tubules of damaged nephrons.

### MiR-21 can be blocked by using LNA-antimiR-21

In order to investigate the influence of miR-21 on kidney regeneration, we wanted to inhibit up-regulation of this miRNA after renal injury. To achieve this, we made use of a specific LNA-antimiR-21 oligomer. These stabilized RNA-oligonucleotides have been shown to bind specifically to their target miRNA and prevent them from binding to their respective mRNA targets [23, 24]. The antimiR-21 was injected intraperitoneally in two different concentrations (10 μg and 20 μg/g body weight) into *N. furzeri* and different organs were analyzed for detectable miR-21 levels (Fig. 2e)*.* The most efficient blocking of miR-21 was detected in kidney and liver, where a high amount of blood is filtered. Levels of miR-21 in the kidney were decreased very efficiently after administration of antimiR-21 at both concentrations. Similar results were received for the liver with a more pronounced dose-dependence. A clear inhibition of miR-21 was also seen in the skin. A decrease of miRNA-level of more than 3-fold was found with both concentrations. In the brain no miR-21 reduction was observed, presumably due to an inability of the antimiR-21 to cross the blood brain barrier. Based on this experiment, 10 μg/g body weight was used in the following experiments as standard concentration. Before performing regeneration kinetics, we tested the stability of antimiR-21 in the kidney (Fig. 2f). A single dose was injected and tissue was collected at different time points over a period of 15 days. Analysis of miR-21-levels revealed that it is efficiently blocked by the antimiR-21 in the kidney of *N. furzeri*, even after 15 days. To examine whether miR-21 inhibition influences expression of other miRNAs, we measured *miR-30a* and *miR-194* levels in antimiR-21 injected fish at distinct time points after kidney damage (Fig. 2g and h). In samples injected with antimiR-21, *miR-30a* showed no difference in expression levels, compared to control. For miR-194, down-regulation was observed in control and antimiR-21 treated fish, but no significant differences were observed between both groups. These data show that antimiR-21 did not influence expression of these two miRNAs and can thus be considered specific.

### Inhibition of miR-21 leads to delayed regeneration

**Fig. 3:**
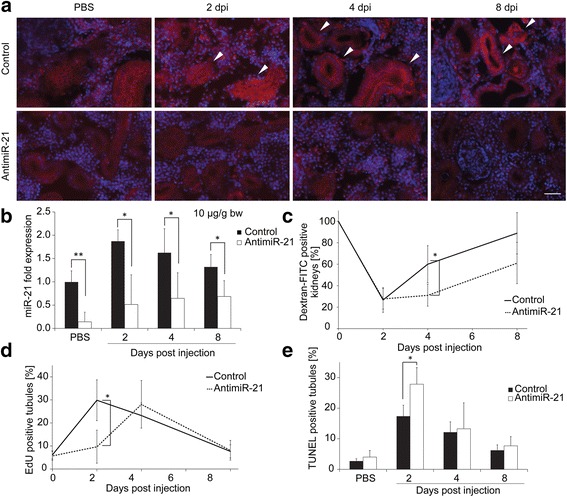
Inhibition of miR-21 up-regulation after renal damage leads to a delay in tubular regeneration. **a** *In situ* hybridization of control und antimiR-21 treated kidney samples after administration of gentamicin. White arrows indicate miR-21 positive tubules in control kidneys. Scale bar: 20 μm **b** Quantification of miR-21 expression by qPCR in control and antimiR-21 treated samples after administration of gentamicin. Hs-RNU1A-11 was used for normalization. **c** Comparison of renal recovery of control and antimiR-21 treated kidneys after damage. Dextran-FITC positive kidneys were count and related to number of all kidneys in the respective groups. *n* = 12 fish/time point **d** Kinetics of proliferation after kidney damage. Proliferation was measured by injection of EdU 2 h before fish were sacrificed and number of EdU positive tubules on sections was counted for every sample. *n* = 4 fish/time point **e** Kinetics of apoptosis after kidney damage. Apoptosis was determined by counting TUNEL-positive tubules, which were related to total number of tubules. *n* = 4 fish/time point, student’s *t*-test was used: *p < 0.05, **p < 0.01

MiR-21 up-regulation was blocked by injection of a single dose of antimiR-21 6 h prior to induction of renal injury by gentamicin. We performed *in situ* hybridization and qPCR to assess whether inhibition of miR-21 was successful. In samples treated with gentamicin and mismatch control, an up-regulation in the tubules was observed after kidney damage by *in situ* hybridization (Fig. 3a). In samples treated with the antimiR, no miR-21 up-regulation was found. These data were confirmed by qPCR (Fig. 3b). We concluded that the antimiR-21 was working effectively and should therefore prevent binding of miR-21 to its target mRNAs. To examine whether this had an impact on regeneration, different parameters were measured. The functional recovery of the kidney was determined by injection of dextran-FITC (Fig. 3c). At 2 dpi, antimiR-21 injected and control fish both were largely unable to reabsorb dextran-FITC (73 % control vs. 72 % antimiR-21). When comparing control and antimiR-21 treated samples at 4 dpi a significant delay in functional recovery was observed. While 60 % of control fish were able to reabsorb dextran-FITC, only 31 % of antimiR-21 treated fish showed green fluorescence in the kidney. This difference was seen also at 8 days post injection. MiR-21 is known to be pro-proliferative [25]; therefore, proliferation of cells in renal tubules was determined by injection of EdU (Fig. 3d). While in fish treated with mismatch control, an increase in tubular cell proliferation was seen at 2 dpi blocking of miR-21 led to a delay of proliferation by 2 days. We also measured apoptosis in renal tubules and found that miR-21 inhibition caused more cells to go into apoptosis (Fig. 3e). Taken together, these findings suggest that *miR-21* up-regulation positively influences initiation of regeneration in fish kidney. Blocking miR-21 leads to a functional delay in regeneration, as seen in a shifted proliferation peak and more apoptosis in renal tubules.

### AntimiR-21 treatment changes gene expression patterns

**Fig. 4:**
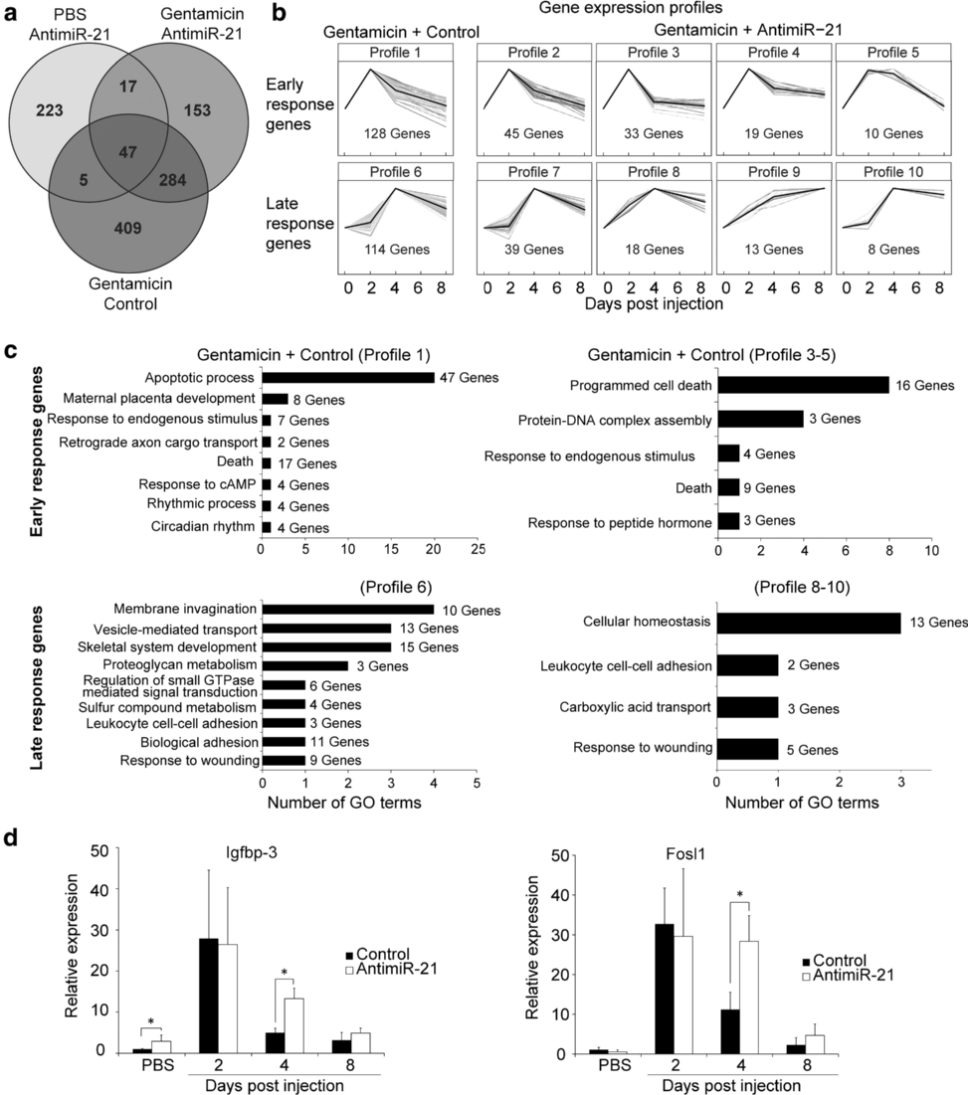
RNA-Seq analysis of control and antimiR-21 treated fish reveals changes in expression profiles. **a** Venn diagram showing number of DEGs for the different samples (*P* < 0.01). **b** The two most significantly enriched expression profiles comprised up-regulated DEGs (early response on top, late response below). Profile 1 and 6 show DEGs for gentamicin/mismatch control treatment, while correlated behavior of same genes after gentamicin/antimiR-21 treatment is displayed in profiles 2 to 5 and 7 to 10. **c** GO-term enrichment analysis of genes from B. Displayed is the number of enriched GO terms per representative similarity group term as well as the number of unique genes supporting the respective terms. **d** qPCR analysis of *ifgbp3* and *fosl1*; student’s *t*-test was used: *p < 0.05, *n* = 5-6 fish/time point

In order to identify genes that might be deregulated by antimiR-21 administration and thus explain the observed changes in regeneration we performed RNA-Seq. We used kidney samples from four groups at four different time points, namely at 0, 2, 4 and 8 dpi. As control we considered kidney RNA from fish that had been injected with a mismatch LNA. To assess the effect of gentamicin on gene expression we used RNA from fish that had received gentamicin together with the mismatch oligonucleotide. A third group got injected with the antimiR-21 together with gentamicin. The fourth group comprised animals injected with antimiR-21 only. Subsequently, we identified significant differentially expressed genes (DEGs) (Fig. 4a and Additional file 2: Table S1). Given that miRNAs in general have a modulatory role and do not serve as on/off switches, it is not surprising that gentamicin had a more significant effect on gene expression (745 DEGs) than the application of the antimiR-21 (292 DEGs). We subsequently focused on genes that were differentially regulated between gentamicin/mismatch control and gentamicin/antimiR-21 injection. We performed cluster analysis using STEM and identified clusters with up-regulation of gene expression at either 2 (early response) or 4 dpi (late response) as most significantly enriched patterns (Fig. 4b). In case of gentamicin/mismatch control 128 genes were observed to be up-regulated in early response (Profile 1 in Fig. 4b). Of those 45 genes maintained their expression upon gentamicin/antimiR-21 administration while 62 of the genes showed an altered expression profile after injection of antimiR-21 (Profile 3 to 5). From 114 DEGs of the late response upon gentamicin/mismatch injection (Profile 6), 39 genes displayed a changed expression profile after gentamicin/antimiR-21 treatment (Profile 8 to 10). GO-term enrichment analysis for DEGs from gentamicin/mismatch injection ‘early response’ (profile 1) revealed ‘apoptotic process’ as term comprising most DEGs (Fig. 4c). The most notable changes after gentamicin/antimiR-21 injection were observed in the category ‘programmed cell death’. This is in agreement with the observed enhanced apoptosis upon antimiR-21 treatment (Fig. 3e). GO-term analysis for DEGs at the later time point revealed terms such as ‘membrane invagination’ (profile 6) and ‘cellular homeostasis’ (profiles 8 to 10) pointing to reorganization of cells. A common term between the gentamicin/mismatch control and gentamicin/antimiR-21 samples is ‘response to wounding’ suggesting that genes affecting these processes are affected by gentamicin and display altered expression upon antimiR-21 administration. We selected two genes for further analysis (Fig. 4d): insulin-like growth factor-binding protein-3 (*igfbp-3*), which shows a profile 3-like expression pattern and FOS-like antigen 1 (*fosl1*) from profile 5. Quantitative PCR showed that while gentamicin treatment influenced expression of both genes, this was significantly changed after antimiR-21 treatment, particularly at 4 dpi. Of note, *igfbp-3* has been linked to apoptosis and oxidative stress in the kidney [26] and was recently identified as miR-21 target gene in glioblastomas [27]. *Fosl1* belongs to the AP-1 transcription factor complex, leading to cellular growth [28]. Recent results indicate that the AP-1 complex could be a direct target of miR-21 as well [29].

## Conclusion

In summary, our data suggest a novel role for mir-21 in kidney regeneration. Among the genes that might mediate its function are *igfbp-3* and *fosl1*. MiR-21 seems to be required for initiating fast proliferation after damage as its knockdown delays proliferation. A similar phenomenon has been described in mouse liver regeneration [23]. Our functional and transcriptome analyses also suggest that in the context of kidney regeneration miR-21 acts as an anti-apoptotic factor. This has also been found to be the case in an ischemia-reperfusion injury model in the mouse [30]. In addition our data show that miR-21 can act differently in the same organ in different vertebrate species, as no significant fibrosis is observed in fish.

## Methods

### Animal experiments

Fish at the age of 16 weeks of the strain MZCS 08/122 [31] were anesthetized and injected intraperitoneally with 10 μg/g body weight (bw) LNA-antimiR-21 or mismatch control (antimiR-21 CCA ACA CCA GTC TGA TAA GCT/3CholTEG; antimiR-21_mismatch control ACC ACA CTA GAC TGC TAA GAT/3CholTEG) and gentamicin (200 μg/g bw). Dextran-FITC was injected 24 h before killing. RNA was prepared from organs and kidney was used for immunohistochemistry. All animal experiments were performed according to the “Principles of laboratory animal care” and to current version of the German Law on the Protection of Animals.

### In situ hybridization

LNA *in situ* hybridization was done on kidney cryosections according to a published protocol [32] using the detection probes from Exiqon (dre-miR-21 miRCURY LNA Detection probe /5DigN/GCC AAC ACC AGT CTG ATA AGC TA/3Dig_N/) in a concentration of 7.5 pMol.

### RNA isolation, cDNA synthesis and qPCR

RNA was isolated from kidney using TRIzol (Thermo Fisher Scientific). cDNA was generated using the iScript ™ cDNA Synthesis Kit (BioRad) and 500 ng total RNA. To generate cDNA from miRNA the miScript®II RT Kit (Qiagen) and 500 ng total RNA was used. qPCR was performed using the CFX384 Touch™ Real-Time PCR Detection System (BioRad) and the miScript SYBR® Green PCR Kit (Qiagen). Each sample was measured in triplicates. Specific forward primers were ordered from Qiagen (miScript Primer Assays) and the reverse universal primer was provided in the miScript ® II RT Kit. Primers: miR-21 UAG CUU AUC AGA CUG GUG UUG GC; miR-30a UGU AAA CAU CCU CGA CUG GAA G; miR-200a UAA CAC UGU CUG GUA ACG AUG U; miR-194a UGU AAC AGC AAC UCC AUC UCC A; igfbp-3_for CTG CAG GTC AGG TGC GAA CGG A; igfbp-3_rev AGC GCG CAC GTC ATG CAG CAG; fosl1_for TTG GCA GCA GCA AAG TGT CGT AAT CGT; fosl1_rev GGA CGA TGA GCT TCC AGA ACC AAT TCA A.

### Immunohistochemistry

Fish were injected with EdU (10 μg/g bw) 2 h before being sacrificed. Kidneys were prepared and embedded in paraffin. Click-iT® EdU Alexa Fluor® 488 Imaging Kit (Life Technologies) was used for detection. Apoptotic cells were identified using the In situ Cell Death Detection Kit, TMR Red (Roche). Paraffin sections were deparaffinized, re-fixed for 15 min and washed in PBS. Subsequently, proteinase K digestion (20 μg/ml) was performed, followed by re-fixation and two washing steps. The enzyme mix was pipetted on slides and incubated for 60 min at 37 °C in the dark, followed by washing and mounting.

### RNA-Seq and bioinformatics

Total RNA was quality checked and quantified using Agilent Bioanalyzer 2100 and Agilent RNA 6000 nano kit (Agilent Technologies). The average RNA integrity number (RIN) of the samples was 9.6 with a minimum value of 9. Around 1 μg of total RNA was used for library preparation employing Illumina’s TruSeq RNA sample prep kit v2 following the manufacturer’s description. This procedure contained selection of polyA RNA species, chemical fragmentation and reverse transcription using random hexamers. The libraries were again quality checked and quantified using the Bioanalyzer 2100 and Agilent’s DNA 7500 kit. Sequencing was done on the HiSeq2500 (Illumina) in high-output, 50 nt single-read mode. The libraries were multiplexed with a factor of six per lane. Reads were extracted in FastQ format using bcl2fastq v1.8.4 (Illumina). Sequencing resulted in around 30mio reads per sample.

Reads were mapped to the *Nothobranchius* transcriptome [33] using bowtie [34]. Reads per gene/transcript were subsequently counted. Gene expression analysis was carried out with edgeR [35] and DESeq [36]. STEM [37] was used for expression pattern profiling. Zebrafish orthologues for *Nothobranchius* genes were retrieved using Blast. Subsequently, human orthologues were fetched with R package orthology [38]. GO enrichment analysis was carried out using DAVID [39] and summarized by REVIGO [40] (0.5 allowed similarity, Homo sapiens GO term sizes, SimRel measure).

## Additional files

Additional file 1: Figure S1. Trichrome staining of kidney samples treated with antimiR-21 at different time points after gentamicin injection. As positive control old fibrotic fish kidney was used. Blue indicates fibrotic tissue, hematoxylin and eosin was used as counterstain. (PPTX 67880 kb) Additional file 2: Table S1. Clustered differentially expressed genes after treatment of N. furzeri with gentamicin, antimir-21 orgentamicin/antimir-21. (XLSX 429 kb)

## Abbreviations

AP-1: Activator protein 1
DEG: Differentially expressed gene
EdU: 5-ethynyl-2'-deoxyuridine
FITC: Fluorescein isothiocyanate
Fosl1: Fos-related antigen 1
GO-term: Gene Ontology-term
H&E: Hematoxylin and eosin staining
Igfbp-3: Insulin-like growth factor-binding protein 3
LNA: Locked nucleic acid
miRNA: MicroRNA
qPCR: Quantitative real time polymerase chain reaction
TUNEL: Terminal deoxynucleotidyl transferase dUTP nick end labeling
