# Supplementary material for: MiR-21 is required for efficient kidney regeneration in fish
Source: BMC Dev Biol. 2015 Nov 17;15:43. doi: 10.1186/s12861-015-0089-2 (PMC4650918; doi:10.1186/s12861-015-0089-2)
Supplement: Additional file 1: Figure S1. — Trichrome staining of kidney samples treated with antimiR-21 at different time points after gentamicin injection. As positive control old fibrotic fish kidney was used. Blue indicates fibrotic tissue, hematoxylin and eosin was used as counterstain. (PPTX 67880 kb) [file 12861_2015_89_MOESM1_ESM.pptx]

## Slide 1
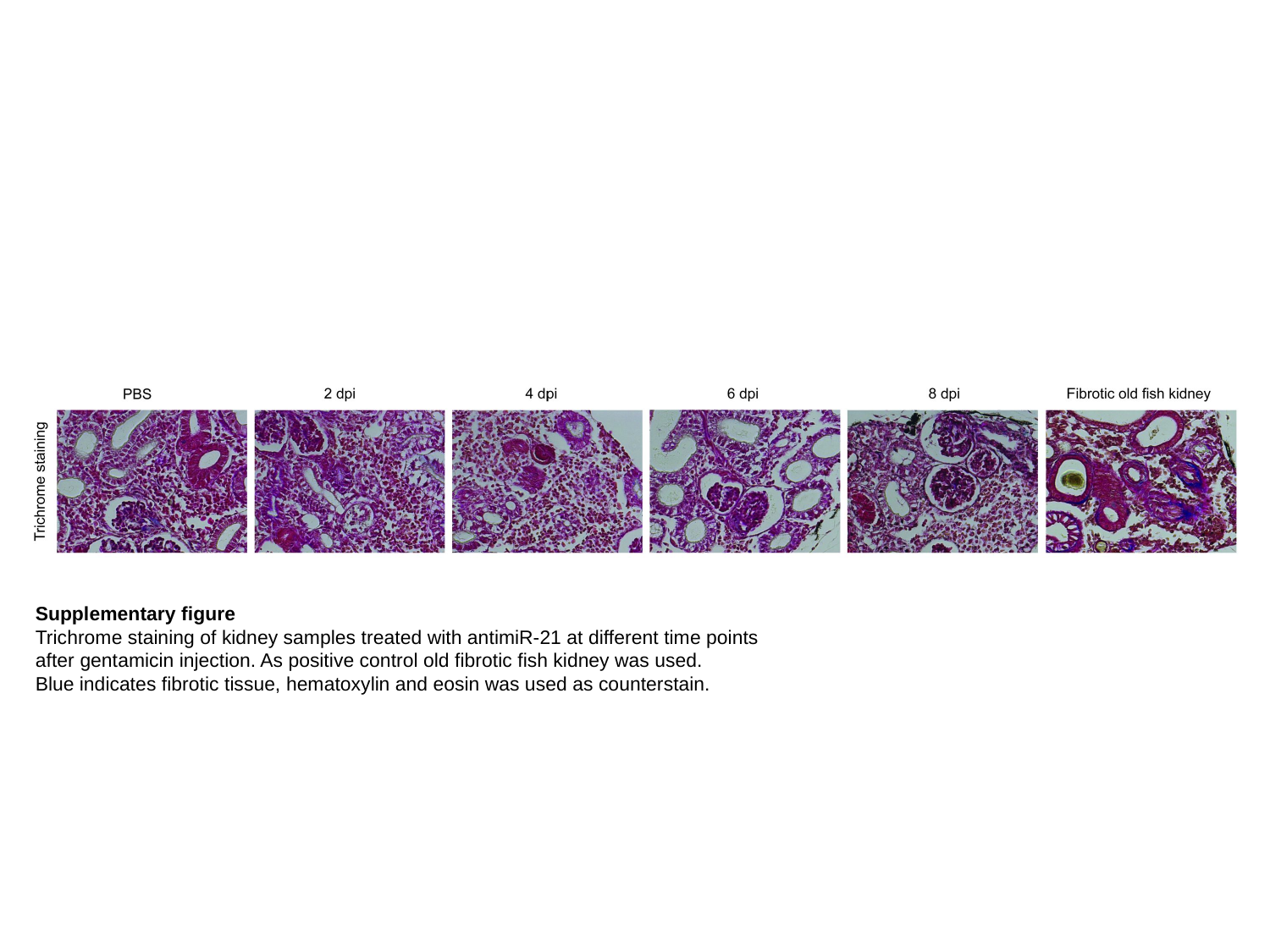

Supplementary figure
Trichrome staining of kidney samples treated with antimiR-21 at different time points
after gentamicin injection. As positive control old fibrotic fish kidney was used.
Blue indicates fibrotic tissue, hematoxylin and eosin was used as counterstain.
